# The prion hypothesis in Parkinson's disease: Braak to the future

**DOI:** 10.1186/2051-5960-1-2

**Published:** 2013-05-08

**Authors:** Naomi P Visanji, Patricia L Brooks, Lili-Naz Hazrati, Anthony E Lang

**Affiliations:** 1 Division of Patient Based Clinical Research, Toronto Western Research Institute, and the Edmond J. Safra Program in Parkinson’s Disease, Toronto Western Hospital, McLaughlin Pavilion, 7th Floor Rm 7-403, 399 Bathurst Street, Toronto, Ontario M5T 2S8, Canada; 2 Tanz Centre for Research in Neurodegenerative Diseases, University of Toronto, 6 Queens Park Crescent West, Toronto, Ontario M5S 3H2, Canada

**Keywords:** Prion, Parkinson's disease, Braak staging, Alpha synuclein

## Abstract

Parkinson’s disease (PD) is a progressive neurodegenerative disorder typified by the presence of intraneuronal inclusions containing aggregated alpha synuclein (αsyn). The progression of parkinsonian pathology and clinical phenotype has been broadly demonstrated to follow a specific pattern, most notably described by Braak and colleagues. In more recent times it has been hypothesized that αsyn itself may be a critical factor in mediating transmission of disease pathology from one brain area to another. Here we investigate the growing body of evidence demonstrating the ability of αsyn to spread transcellularly and induce pathological aggregation affecting neurons by permissive templating and provide a critical analysis of some irregularities in the hypothesis that the progression of PD pathology may be mediated by such a prion-like process. Finally we discuss some key questions that remain unanswered which are vital to determining the potential contribution of a prion-like process to the pathogenesis of PD.

## 

### Braak staging of Parkinson’s disease

Braak and colleagues devised their widely accepted staging system of PD progression by assessing the regional distribution of αsyn immunoreactive structures in the brains of 110 αsyn positive subjects (69 incidental and 41 clinically diagnosed PD patients). According to the Braak model, the disease process commences in the lower brainstem in the dorsal motor nucleus of the vagus nerve (DMV), as well as anterior olfactory structures. The disease then ascends rostrally from the DMV through susceptible regions of the medulla, pontine tegmentum, midbrain, and basal forebrain, eventually reaching the cerebral cortex (Figure 1) [1]. This is a non-random, progressive process, with specific nuclei and neuronal types giving rise to the development of Lewy pathology in a stereotypic pattern. As the pathology advances upwards from the brainstem, both the severity of the lesions and the clinical manifestations of the disease increase [2].

**Figure 1:**
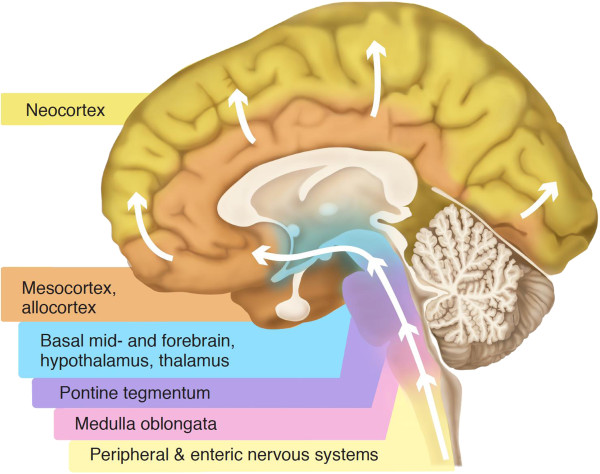
**Staging of Lewy pathology according to the Braak model.** Schematic summarizing the progression of Parkinson’s disease as proposed by Braak and colleagues [1]. According to the Braak model, αsyn deposits in specific brain regions and neuronal types giving rise to Lewy pathology in a stereotypic, temporal pattern that ascends caudo-rostrally from the lower brainstem (including the dorsal motor nucleus of the vagus nerve in the medulla then the coeruleus-subcoeruleus complex, raphe nuclei, gigantocellular reticular nucleus in the medulla aand pons) through susceptible regions of the midbrain (substantia nigra and the pedunculopontine tegmental nucleus) and forebrain (e.g., amygdala) and into the cerebral cortex (e.g., anteromedial temporal mesocortex, cingulate cortex and later neocortical structures). It is hypothesized that the disease initiates in the periphery, gaining access to the CNS through retrograde transport along projection neurons from the gastrointestinal tract. As the disease progresses, the severity of lesions in the susceptible regions increases.

The Braak model of PD staging hinges on the notion that Lewy pathology is not random: vulnerable sites in the brain are affected in a predictable topographic sequence (Figure 1). In support of this, Braak and colleagues have demonstrated that the cell types in the central nervous system (CNS) exhibiting a propensity for developing Lewy pathology share common features. Specifically, projection neurons with a long, thin, unmyelinated or poorly myelinated axons are particularly susceptible to developing lesions [3,4]. In agreement with this, cortical and subcortical projection neurons giving rise to long, sturdy, heavily myelinated axons appear to be resistant to pathology [3]. According to the Braak model, it is this apparent selective vulnerability of specific neuronal types that accounts for the predictable regional spread of Lewy pathology observed in PD.

Although the Braak model outlines a pathogenic process that initiates in the brainstem and advances rostrally, it is unclear if the brainstem is the site of the initial disease process. Indeed it is known that PD extends beyond the boundaries of the CNS, affecting the peripheral nervous system (PNS), and in particular the enteric nervous system (ENS). This largely stems from the clinical observation that PD patients frequently have a wide range of non-motor symptoms related to dysfunction of the PNS, which often precede the motor symptoms of PD. In support of this, Lewy pathology is found throughout the PNS in both clinically diagnosed PD patients [5] as well as cases of clinically asymptomatic incidental Lewy body disease (ILBD) [6-8]. The finding of Lewy Bodies in the brains of asymptomatic individuals without parkinsonism (i.e., ILBD) is of particular interest as these cases are thought to represent presymptomatic PD in the very beginning stages of the disease process [7].

Braak and colleagues have put forth the notion that the pathogenic process of PD begins when an environmental insult enters the body and subsequently gains access to the CNS, where it then spreads trans-synaptically from one vulnerable brain region to the next [3,9]. This “dual-hit” hypothesis postulates that the unidentified neurotropic pathogen enters the brain through both a nasal and a gastric route. The nasal route is used to explain the early involvement of olfactory structures, as well as the olfactory dysfunction that is common in early PD [3,9]. In support of this, Lewy pathology is found in both the anterior olfactory nucleus as well as olfactory bulb mitral cells, the projection neurons that receive input from the olfactory epithelium [1,10].

Despite the initial involvement of the olfactory system, Braak *et al*. [3] do not consider olfactory structures to be the point of departure for Lewy pathology to the rest of the brain. Instead, they propose that the unknown pathogen gains access to the brain through the gastric system. The gastric route would fit with the early involvement of the ENS in PD. Like olfactory impairment, gastrointestinal dysfunction is common in PD and seems to emerge early in the disease course [11], often preceding clinical parkinsonism by many years [12]. Lewy pathology has long been known to occur in the gastrointestinal tract of PD patients and is well-documented in early, mid- and late PD [13-17]. Its presence in the premotor phase of the disease has been supported by a recent study of three patients who were found to have αsyn staining in bowel biopsy samples obtained 2-5 years before they presented with signs of PD; such staining was not seen in 23 healthy controls [16]. In accordance with the Braak model, the cells in the ENS that are vulnerable to Lewy pathology share the key characteristics of those vulnerable in the CNS (i.e. projection neurons with a long, thin, unmyelinated axon) [18]. If the ENS is the site of induction for a potential PD-causing agent, a pathogenic insult entering the body via the gastrointestinal tract must then gain access to the CNS. The visceromotor projection neurons of the DMV give rise to preganglionic fibers that innervate the ENS. Braak *et al*. [3] have proposed it is these unmyelinated vagal preganglionic neurons that could provide the route for a pathogen to be retrogradely transported from the ENS into the CNS. This notion is supported by the observation that these vagal projection neurons are some of the first cells in the brain to display Lewy pathology [3]. It may therefore be that a pathogenic agent enters the body and gains access to the gastrointestinal tract, invades vulnerable neurons in the ENS and is subsequently retrogradely transported to the CNS through vagal preganglionic fibers. Indeed, two recent reviews have proposed a similar mechanism by which misfolded proteins in a host of neurodegenerative diseases may spread from cell-to-cell within the ENS eventually reaching the CNS [19,20]. Thus, within the ENS, misfolded proteins, including αsyn, undergo spontaneous glycation to form advanced glycation end products (AGEs), which are excreted via exosomes. Once in the extracellular space, AGEs are recognized by cell surface receptors for advanced glycation end products (RAGEs) which act to internalize the misfolded AGE. AGE-RAGE binding activates multiple intracellular signaling pathways including a positive feedback loop whereby there is an increased cell surface expression of RAGE along with induction of oxidative stress and inflammation. Through this positive feedback, there is cell-to-cell spreading of disease throughout the enteric wall, and perhaps beyond. In the context of Parkinson’s disease, it is proposed that once in the DMV, the pathogen follows an ascending course, spreading from one susceptible cell group to the next (Figure 1).

### Parkinson’s disease as a prion disorder

#### Transplanted dopamine neurons in humans

The Braak model puts forth evidence for the progressive, stereotypic spread of an unknown pathogenic insult from one affected brain region to the next. Building on this, there has been considerable interest in recent years in the possibility that this disease progression is mediated in a prion-like fashion, with the spread and seeding of sequentially involved brain areas by misfolded proteins. The suggestion that PD may be a prion disorder stems from the observation of Lewy body pathology in embryonic dopamine neurons transplanted into the putamen of human PD patients [21-24]. In four separate case reports, autopsies performed 10-22 years post-transplantation found evidence of αsyn-positive, Lewy-body-like inclusions in grafted human dopamine neurons. Such inclusions are not normally seen in neurons of such a young age. These inclusions stained positively for thioflavin-S (indicating the presence of β-sheets) and ubiquitin and the affected transplants showed reduced dopamine transporter and tyrosine hydroxylase levels. In concert with the discussed evidence of a sequential staging of PD pathology, these observations lead to the proposal that the progression of PD may be mediated by a prion-like process [25,26]. Furthermore, in contrast to Braak *et al*.’s thinking, the proposal was made that αsyn itself may be the pathogenic factor underlying the spread of the disease. Adding weight to this proposal, similar prion-like mechanisms have been proposed for other proteins involved in several other neurodegenerative diseases, including Alzheimer’s, Huntington’s and amyotrophic lateral sclerosis [27-29].

#### In vitro evidence

Prions are composed of the PrPsc protein, a misfolded form of endogenous PrPc, and underlie disorders such as Creutzfeldt-Jakob disease, bovine spongiform encephalopathy and scrapie [30]. The conversion of alpha helical PrPc to the β-sheet rich PrPsc confers an infectivity that is the defining feature of a prion [31]. Indeed, as all other known infectious agents contain either DNA or RNA, the term prion, derived from the words proteinaceous and infectious, was given to this unique PrPsc protein [32]. Though the precise molecular mechanisms underlying the propagation of the prion protein are unknown, it is universally accepted that PrPsc acts as a template upon which native PrPc is refolded into PrPsc. Extensive *in vitro* studies have shown that, when incubated at 37°C, monomeric αsyn forms fibrils reminiscent of those contained in Lewy bodies in PD [33-35]. Furthermore, existing fibrils can act as seeds, promoting fibrillization of surrounding monomeric αsyn [36]. Closer inspection of the nature of the seed-induced fibrils has revealed that addition of seeds produced from αsyn bearing the A30P mutation to wildtype monomeric αsyn leads to the generation of fibrils with the same character as A30P fibrils [37]. Thus it would appear that the assembly of wildtype fibrils induced by A30P seeds involves a conformational change in wildtype αsyn to that of A30P. This process is remarkably similar to that described for the templated conversion of PrPc to PrPsc. In the same study it was noted that the addition of wildtype seeds to monomeric A30P αsyn did not involve a conversion to the wildtype fibril. This feature of templated conversion seems to apply to the A30P αsyn mutation responsible for a small number of familial cases of PD but not to the wildtype form of αsyn responsible for the majority of cases of PD. Thus, in the context of this review regarding PD we would define a prion-like mechanism of disease propagation as a mechanism by which native protein with rich α-helical structure is refolded into a toxic form with high β-sheet structure triggering the misfolding of further native protein in a self-perpetuating process. A form of the protein capable of triggering this process can be conferred from one cell to another, thus inducing progressive neurodegeneration.

Beyond the test tube, a series of *in vitro* tissue culture experiments have demonstrated the following features that make αsyn an attractive candidate as the pathogenic factor implicated in the prion-like spread of PD pathology: 1) Fibrillar αsyn has been shown to be transported via axonal transport [38]. 2) Monomeric and aggregated forms of αsyn, contained in cytosolic vesicles, are released from neurons by an unconventional exocytotic mechanism [39]. 3) Aggregated forms of αsyn (fibrillar and non-fibrillar oligomers) are taken up into neurons via conventional endocytosis, whereby they can move through the endosomal pathway and are eventually degraded by the lysosome [40]. 4) Transduction of preformed fibrillar αsyn into cells overexpressing αsyn results in the formation of insoluble intracellular inclusions recapitulating several key features of Lewy bodies including size, subcellular localisation, β-pleated sheet conformation, and hyperphosphorylation and polyubiquitination of constituent αsyn [41]. Furthermore, using C-Myc tagged preformed αsyn fibrils, it has been demonstrated that soluble endogenous αsyn becomes recruited to these insoluble intracellular inclusions eventually comprising the major component [41]. Several other studies have employed co-cultures in a wide variety of cell lines to demonstrate the ability of exogenous αsyn, be it monomeric, oligomeric or fibrillar, to infiltrate surrounding cells and induce a pathological aggregative response [42,43]. These cellular mechanisms, by which αsyn can be both released from and taken up by neurons, are illustrated in Figure 2. Thus, at the *in vitro* level there is compelling evidence that αsyn is capable of spreading an aggregation-inducing disease-like pathology from one neuron to another.

**Figure 2:**
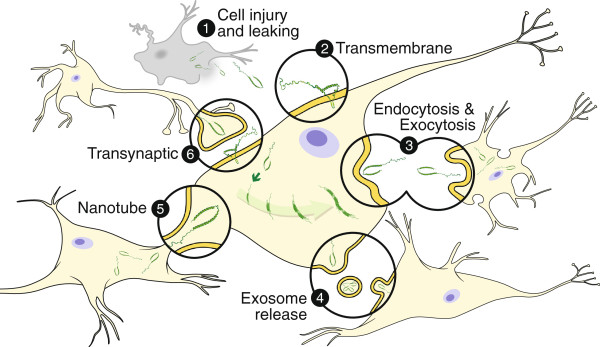
**Potential mechanisms of neuron-to-neuron transmission of synuclein.** Alpha synuclein can be released into the extracellular space via (1) leakage from injured cells with compromised membrane integrity. Extracellular synuclein could then directly translocate the cell membrane and gain access to neighboring neurons (2). Synuclein can also be transmitted from cell-to-cell via conventional exocytosis and endocytosis (3). Synuclein can be packaged into exosomes which are released and taken up by surrounding cells (4).Tunneling nanotubes can form a direct connection between two cells potentially allowing synuclein to transfer freely from one cell to another (5). Finally, synuclein could be transmitted by direct synaptic contact (6).

#### In vivo studies

More recent studies have investigated the translation of these findings *in vivo*. Desplats *et al*. grafted green fluorescent protein (GFP) labelled mouse cortical neuronal stem cells (MCNSCs) into the hippocampus of mice overexpressing human αsyn [43]. Using double immunofluorescence, these authors demonstrated that seven days post grafting ~2.5% of MCNSCs had taken up αsyn from the host tissue; 28 days post grafting this number had risen to ~15%. Although these data demonstrate host to graft transmission of αsyn *in vivo*, ultrastructural analysis of inclusion bodies within grafted cells found no evidence of the fibril or aggregate formation following infiltration by αsyn that was evident in their *in vitro* studies. A more recent study mimicked more closely the clinical situation grafting murine mesencephalic neurons into the striatum of mice overexpressing human αsyn [42]. Six months post grafting a small number of grafted cells were found to contain small human (i.e., host) αsyn positive punctae. These authors also demostrated that the structural form of αsyn had no effect on the ability for cellular uptake *in vivo*. Thus monomeric, oligomeric and fibrillar recombinant αsyn were all found in neuronal cells following a stereotaxic injection in rats. Interestingly, this phenomenon of αsyn uptake is not limited to neurons, but has also been demonstrated in astrocytes both in cell culture and *in vivo*, whereupon it triggers an inflammatory response in the recipient cells [44]. As inflammation is increasingly implicated in a host of neurodegenerative diseases, this process may represent an alternative means by which αsyn can mediate the pathological progression of PD. A recent study using a transgenic mouse model of synucleinopathy (TgM83), demonstrated that inoculation of young asymptomatic mice with brain homogenates prepared from older symptomatic mice accelerated the presence of both αsyn hyperphosphorylated at serine 129 and aggregated αsyn and significantly decreased survival time [45]. Furthermore, this hastened pathology was absent in αsyn knockout animals inoculated with the same brain homogenates prepared from older symtomatic transgenic mice. This suggests a strong role for endogenous host αsyn in the transmission of pathology from affected to unaffected sites and is consistent with a prion-like mechanism of disease propagation.

In the aforementioned studies there was no evidence of a spread of pathologic αsyn to more widespread areas of the brain distal to the sites of application. However, two recent studies have expanded upon this. Angot et al., (2012) used a viral vector (AAV2/6-hu αsyn) to engineer rat nigral neurons to express human αsyn which, over time, was transported from nigral cell bodies to the terminals in the striatum [46]. These authors then grafted rat ventral mesencephalic neurons into the striatum and observed transmission of human αsyn expressed by the virally transduced nigrostriatal cells into the grafted tissue. Indeed, the extent of transfer of the virally expressed human αsyn was shown to be of a much greater magnitude than that observed in previous studies examining the transfer of αsyn into grafted tissue. Furthermore, human αsyn immunoreactivity within the grafted cells was shown to be surrounded by a strong immunoreactivity for rodent αsyn, suggesting that the human αsyn supplied by the host tissue had induced a seeding effect in the rodent αsyn present in the grafted cells. This study demonstrated for the first time that αsyn can induce a seeding effect *in vivo* following axonal transport along the nigrostriatal pathway. However, a more recent study by Luk *et al*[47] has demonstrated that this phenomenon can be even more widespread. In this study, brain homogenates from aged transgenic mice, containing significant levels of aggregated A53T human αsyn (the first mutation of αsyn discovered in humans), were injected into the neocortex and striatum of younger animals long before pathology normally develops. 30 days post injection some level of αsyn pathology was evident in the vicinity of the injection sites. However, 90 days post injection αsyn pathology, reminiscent of Lewy bodies and surrounded by astrogliosis and microgliosis, was widespread throughout the brain. These inclusions were not apparent in age-matched control animals innoculated with saline or with homogenates prepared from younger animals. The critical role of host αsyn to this process was also demonstrated by the observed lack of pathology following innoculation in αsyn null mice. In addition, detailed examination demonstrated a recruitment of endogenous murine αsyn to the pathological inclusions further supporting a permissive templating prion-like process. Mapping studies illustrated that it was those brain regions with the most neuronal connections to the sites of innoculation that displayed the most severe pathology (frontal cortex and thalamus) and that there was a relative sparing of regions close by but lacking direct neuronal innervation. Indeed, based on evidence from their studies the authors suggest that the distribution of αsyn pathology following innoculation is suggestive of a trans-synaptic spread. This contrasts with the recent *in vitro* findings of Freundt et al (2012), who found that axon to soma transfer of αsyn did not require synaptic contacts [38].

Although there is now considerable *in vivo* evidence of cell-to-cell transmission of αsyn and recruitment of endogenous αsyn to inclusions reminiscent of Lewy bodies, a crucial outstanding issue is establishing whether there is a link between this and the process of neurodegeneration. A recent elegant study has suggested a causative link between progressive Lewy inclusion formation and neurodegeneration of nigral dopaminergic cells with correspoding deficit in striatal dopamine and motor function [48]. The authors demonstrated a clear time and connectivity dependent spread of Lewy body-like pathology following intrastriatal injection of fibrillar αsyn in mice, with expression seen earliest in those brain regions highly innervated by the striatal injection site (cortical layers IV and V, and the olfactory bulb) and latter spread to more distally connected areas (ventral striatum, thalamus, occipital cortex, and commisural and brainstem fibers). Pathology was particularly abundant in the dopaminergic cells of the substantia nigra pars compacta. Intriguingly, at 30 days post injection, approximately 30% of nigral cells contained αsyn-positive inclusions, which was accompanied by negligible cell loss. At 100 days post injection, both the number of cells bearing αsyn-positive inclusions, the total number of nigral cells and striatal levels of dopamine, declined concommittantly. These data strongly suggest that aggregation of αsyn may directly precipitate the loss of nigral dopaminergic neurons that is a fundamental characteristic of PD.

### Challenges to reconciling Braak staging with a prion-like spread of pathology in PD

Clearly there is increasing evidence, both experimental and clinical, demonstrating that exogenously applied αsyn can infiltrate surrounding cells and initiate a PD-like pathological response. However, it remains to be shown what initiates this process and whether it can account for disease progression in the human brain. Here we evaluate the prion hypothesis of PD progression taking into account the current understanding of the neuroanatomy and pathology of PD and highlight some critical remaining questions that need to be addressed before accepting PD as a prion disorder.

The conserved propagation patterns of αsyn pathology described by the Braak model could indicate a prion-like spread of αsyn. According to Braak, vulnerable brain regions are affected in a predictable sequence, progressing in a stereotypical caudal-rostral pattern starting in the lower brainstem. One could hypothesize that pathogenic αsyn is transported to anatomically connected brain regions along the prescribed route of progression, seeding αsyn aggregation in each vulnerable region encountered. Whether the pattern and progression of pathology can be generalized to the predictable, sequential involvement of vulnerable sites as Braak describes, however, remains controversial. The Braak model postulates that Lewy pathology in the lower brainstem is necessary for the later appearance of Lewy pathology in more rostral structures [1]. However, this may be an artifact of their pre-selection of cases on the basis of involvement of DMV. Multiple studies have challenged the Braak staging scheme, reporting cases with inclusions throughout the brain but with preservation of medullary nuclei [8,49-51]. In fact, recent reports suggest that the Braak system fails to classify upwards of 50% of αsyn immunoreactive cases [50,52,53]. The pathological regional heterogeneity between PD cases suggests Braak’s proposed pathway is not the only possible route of spread and pathology may even emerge simultaneously in multiple subcortical and cortical regions. Such cell autonomous protein misfolding would argue against a Braak model of PD staging. One might expect that a cell autonomous process would better explain genetic causes of PD associated with Lewy bodies. However, that the site of initial emergence of disease may well be variable and perhaps multiple (possibly within certain anatomical constraints) leading to a heterogeneous phenotype, does not preclude that a prion-like mechanism still might govern spread of disease through the brain from these multiple and varied departure points.

Challenges also exist for the proposed “gut to brain” spread of Lewy pathology. Rather than suggesting initial involvement of the ENS with subsequent spread to the DMV, Beach et al (2010) have advocated a "brain to gut" direction of transmission given their findings of a rostro-caudal gradient of phosphorylated αsyn in the GI tract and the known richest vagal innervation of the upper gut [5]. The recent report of Del Tredici and Braak (2012) showing evidence for involvement of the spinal cord only after involvement of the brain also raises question about the earliest involvement of at least some ENS regions [54].

Another prerequisite of the Braak model is that the severity of the lesions in the affected brain regions will increase as the disease progresses [1]. It would therefore be expected that the DMV, Braak’s point of departure for Lewy pathology in the brain, would have more severe pathology than the rostral regions said to be affected later in the disease. This, however, is not true in all cases. In one study, upwards of 65% of cases had αsyn loads in the substantia nigra and locus coeruleus that were roughly equivalent to that found in the DMV [55]. Further still, there are reports of severe involvement of the substantia nigra without any prominent DMV pathology [56,57]. This lack of significant αsyn pathology in the DMV compared to more rostral structures cannot be explained by neuronal loss, as these cases had well preserved neurons. For the Braak-prion hypothesis to account for these cases, pathogenic αsyn would have to spread and seed very rapidly to all vulnerable brain regions once the disease process is initiated, which does not fit with the protracted disease course of idiopathic PD. Alternatively, mechanisms underlying seeding and subsequent aggregation and cell death may be dissociated.

One possible explanation for the inconsistencies between the Braak model and the conflicting reports may be that spreading of pathology can occur in both an anterograde and retrograde direction. Braak and colleagues initially hypothesized that while the olfactory system is affected very early in the disease process, olfactory structures are not the point of departure for Lewy pathology to the rest of the brain, despite connections with cortical and subcortical regions [1]. Subsequent observations from a growing number of groups, including Braak and colleagues, however, have suggested that Lewy pathology may indeed progress along olfactory pathways [9,58-60]. Lerner and Bagic [26] subsequently expanded upon this by proposing a complementary hypothesis to the Braak model, in which they emphasize the possibility of anterograde spread of the pathogen through the olfactory tubercle and bulb to the brainstem, in addition to the retrograde spread from the dorsal motor nucleus of the vagus, as described by Braak *et al*. [1]. The addition of this olfactory route of progression may reconcile some discrepancies with the original Braak model. Although, the bi-directional spread of a pathogen may be at odds with the Braak staging scheme, it is not inconsistent with the concept of neuronal spread of a pathogen in a prion-like fashion. Indeed, Lerner and Bagic suggested such a prion-like mechanism may underlie the neurodegenerative process in PD [26].

A further explanation for the reported inconsistencies with the Braak model may be the clinical diversity of PD. Different subtypes of PD may have different underlying pathological patterns. In support of this, Halliday *et al*. [61] found that PD patients with a younger age of onset and a long clinical course had pathology that fit with the Braak model, whereas patients with older onset and shorter disease duration did not. This suggests that clinical phenotype may predict the pattern of pathology that underlies PD. The fact that Braak and colleagues only selected cases in which the DMV was positive for αsyn immunoreactivity may account for some of these apparent discrepancies, as they may have only examined a select subtype of PD. More recent large-scale neuropathological studies tend to include all cases that are positive for αsyn immunoreactivity, regardless of which brain regions were affected, and as a result may include a more diverse profile of PD and its subtypes. It may therefore be that a prion-like spread of αsyn in a pattern predicted by the Braak model does indeed occur in PD but only in certain subtypes of the disease.

A key outstanding issue when considering using Lewy body pathology to define the progression of PD is regarding whether the Lewy body itself is harmful or protective. Traditionally, the presence of Lewy bodies in sites prone to neurodegeneration has supported the dogma that the Lewy body is detrimental to neuronal survival. However, in recent years many studies have suggested that Lewy body formation is a protective cellular mechanism to sequester toxic αsyn aggregates and even aid their degradation by an aggresome-like mechanism [62-65]. In some cases cells containing Lewy bodies have been described as having a healthier morphology than neighboring Lewy body negative cells [66]. Furthermore, *in vitro* studies have demonstrated that cell death can precede Lewy body formation [67] and in PD it has been shown that the majority of cells undergoing apoptosis do not contain Lewy bodies [68]. As the Lewy body may in fact represent a cell that is resisting neurodegeneration, caution must be used in employing it to define disease progression. Indeed, a recent study examining the relationship between nigral cell loss, distribution of Lewy bodies and disease duration in PD found no evidence to support a correlation between Lewy body distribution or density and neurodegeneration [69].

It is vital to keep this issue in mind when also considering the selective susceptibility of certain cell types and specific brain regions to Lewy pathology. While the Braak model describes projection neurons with a long, thin, unmyelinated axons as the cell type that is prone to Lewy pathology [3,4], it is not known why these neurons are susceptible. Although many explanations could be provided to account for differences between the human disease and animal models, it is interesting to note that the recent important study by Luk *et al*. [47], which provides some of the strongest evidence to date for transcellular propagation of αsyn pathology, demonstrated most marked involvement of heavily myelinated projections, including the internal capsule. Furthermore, it is not clear why some regions with Braak’s “vulnerable” cell types are affected whereas many other anatomically connected/neighboring regions with similar cell types are not. Such cryptic susceptibility is further highlighted in Braak’s detailed description of a non-geographical pattern of Lewy pathology in specific subcortical structures in earlier disease versus a more diffuse spreading throughout the cortex in later stages [1]. It therefore seems that αsyn propagates differently according to the type of brain tissue it encounters; however, it is not clear how or why this would be the case.

To further complicate matters, according to the Braak model, the neuronal type prone to developing Lewy pathology in PD is specific to PD and distinguishes PD from other synucleinopathies [3]. Therefore, the prion-like spread of αsyn must discriminate between cell types across different synucleinopathies. Furthermore, while abnormal aggregation of αsyn is the dominant pathological hallmark of synucleinopathies, the nature of αsyn aggregation is distinct between different disorders. For example, PD, Parkinson’s disease dementia (PDD) and dementia with Lewy bodies (DLB) are characterized by αsyn deposits in neuronal Lewy bodies and Lewy neurites, whereas multiple system atrophy (MSA) is defined by abnormal filamentous deposition of αsyn in the nuclei and cytoplasm of both oligodendrocytes and neurons [70-72]. If prion-like αsyn seeding plays an important role in the pathogenesis of neurodegenerative disease then how and why αsyn behaves differently in different diseases must be addressed (see below). In this context, using the Lewy body as a structural marker for the progression of the disease may not be appropriate. For example Kramer et al., have demonstrated that, in contrast to the relatively small number of cortical Lewy bodies, there are a number of small presynaptic αsyn aggregates in DLB, which may account for the significant cognitive impairment in this disease [63]. Similarly, Milber et al found evidence of neuronal dysfunction preceding Lewy pathology in the nigra in PD [73]. These data suggest that synaptic dysfunction can occur without the presence of Lewy bodies *per se* and that perhaps using the Lewy body alone as a marker of pathological progression is not sufficient to predict the spread of disease.

Finally, if the progression of PD can be explained by a Braak-prion-like spread, a critical question that must be addressed is what initiates the process in the first place? Braak’s dual-hit hypothesis postulates that an environmental neurotropic insult is responsible [3,9]. While the Braak model proposes a viral pathogen, a recent study in mice demonstrated that a peripherally administered environmental toxin (i.e. rotenone) may induce a Braak-like spread of αsyn pathology in the CNS [74]. Although this has not been shown in the human condition, such results do support the hypothesis of an environmental insult triggering the Braak’s cascade of pathology seen in PD. What is not clear, however, is how an environmental insult could induce the involvement of systems outside of Braak’s continuous chain of projection neurons that are presumably isolated from the environment. Perhaps one of the most challenging facts to reconcile with this theory is the very early involvement of the cardiac sympathetic nerves seen in PD and in the presumed pre-clinical state of incidental Lewy body disease [75].

### Other challenges to the prion hypothesis

In addition to the issues outlined above, there are critical questions in the defining of PD as a prion disorder that must be addressed. One substantial issue is indeed semantic. As the word prion itself derives from the words “protein” and “infectious”, to define PD as a prion disorder mediated by αsyn, we must demonstrate that αsyn is capable of “infectivity”. It has been suggested that, due to an apparent lack of microbiological transmissibility and propagation within communities, all amyloidogenic proteins capable of permissive templating be termed “prionoids” [27]. It remains possible that difficulties in verifying an infectious nature of αsyn may be due to the length of time required for the process to occur *in vivo.* Indeed in human post mortem studies, Lewy-like pathology was not observed in grafted tissue 18 months post graft [76]. Accumulation was apparent four years post graft, but not until 10-14 years post graft was full Lewy pathology evident [21]. There are likely several features of both the host and the "infectious" agent that influence the cell to cell transfer, effectiveness of permissive templating, and the distribution and nature of the resulting pathology. For example, in classical prion diseases, the biochemical nature of the prion protein PrPsc and the genetic makeup of the host (M/V at codon 129) have major influences on the nature and distribution of the underlying pathology (and as a consequence the clinical manifestations). The same types of processes may influence the distribution of Lewy body pathology in PD and even determine whether the final disease presentation is PD or that of another synucleinopathy. Furthermore, if indeed αsyn is responsible for a prion-like propagation of the disease, understanding the relatively delayed propagation of αsyn pathology post symptom onset, as compared to the rapid process of conventional prion disorders once symptomatic, could inform the development of therapies to slow the progression of both PD and prion diseases.

We have reviewed considerable experimental evidence supporting the premise that αsyn can be released from and enter neighbouring neurons, and induce aggregation of native αsyn *in vitro*. However, there are some caveats to these experimental settings that necessitate caution in extrapolating these data to the human brain in PD. Firstly, not all tissue culture studies find that supplying exogenous αsyn in the surrounding media is sufficient for infiltration into cells. Indeed in order to get meaningful internalisation Luk and colleagues had to make use of cationic liposomes to facilitate entry of αsyn into the cell [41]. A further caution is required in interpreting the significance of data demonstrating the transmission of αsyn in animal studies employing mice genetically engineered to overexpress αsyn, thus presumably enhancing any host-to-graft transmission, although more recent work demonstrates similar changes in non-transgenic animals [48]. Furthermore, the applicability of animal studies employing direct stereotaxic application of αsyn to the brain to the initiation and propagation of idiopathic PD remains to be shown. Although the recent results of Luk and colleagues in non-transgenic animals [48] are impressive and very promising, it must be admitted that the relevance of using very high concentrations of preformed artificial αsyn fibrils to human PD is unknown. Thus, further studies are required to demonstrate unequivocally that enough αsyn can be released and infiltrate surrounding tissues, by physiological means, to induce aggregation *in vivo.* Secondly, there is controversy in the literature regarding the structural assembly of αsyn required to induce inclusion formation. In the many studies employing co-culture and those stereotaxically implanting cells overexpressing αsyn, the structural nature of the infiltrating αsyn is unclear [42,77]. Tissue culture studies have shown that monomeric, oligomeric and fibrillar αsyn can be taken up by cells [42]. Thus, in co-culture experiments, and those stereotaxically implanting cells overexpressing αsyn, it could be any of these structural forms leading to the aggregate formation within the host. It should also be emphasized that much of this discussion hinges on the concept that the insoluble fibrillar protein aggregates are responsible for cell death, whereas there is increasing evidence implicating soluble oligomers as potential culprits in the pathogenesis of neurodegenerative diseases such as PD [78]. Thirdly, the degradation of different assemblies of internalised αsyn requires further evaluation since some cell culture studies suggest that exogenously applied monomeric αsyn is quickly degraded by the lysosome whereas fibrillar αsyn endures [41]. Conversely, other cell culture studies have found that oligomeric αsyn is degraded by the lysosome, and that monomeric αsyn rapidly and directly translocates to cellular membranes thus resisting lysosomal degradation and is therefore the more likely disease spreading form [40]. Finally, although the recent studies by Angot *et al* and Luk *et al*. [46,47] have demonstrated a putative trans-synaptic spread of αsyn pathology *in vivo*, crucially, the transfer of αsyn through the neuroanatomical pathways implied in Braak’s staging scheme has not yet been shown. Indeed in the study of Luk *et al*. [47], stereotaxically applied αsyn was demonstrated to travel in both an anterograde and retrograde manner, being evident in both the thalamus and the substantia nigra pars compacta following a striatal application in mice. As mentioned above, such a bidirectional spread seems at odds with Braak staging in PD, although does not argue against a prion-like disease mechanism. Future *in vitro* studies should be performed to investigate the ability of αsyn applied in the ENS or olfactory bullb to spread along the pathways described by Braak staging.

The occurrence of Lewy bodies in transplanted fetal nigral neurons in patients with PD has been a strong incentive to consider a prion-like spread of pathology. However, not all case reports find pathology in grafted tissue [79]. In those that do, Lewy bodies are present “within a minority” of grafted cells [22] although it should be noted that the proportion of transplanted neurons reported to contain Lewy bodies is almost identical to the proportion of Lewy body positive neurons in the substantia nigra in Parkinson’s disease. Despite this, it must be acknowledged that Lewy bodies contain much more than aggregated αsyn and occur in many disorders in which αsyn is not believed to be a primary contributor to the overall pathogenesis. A more plausible explanation may be that multiple other toxic factors that are known to exist in the PD brain, such as proinflammatory cytokines, reactive oxygen species (supported by the extent of neuromelanization uncharacteristic of such young neurons [21]) and pro-apoptotic factors, may damage the cell leading to a disruption of cellular processes and explain the presence of Lewy bodies in previously healthy grafted dopaminergic cells. This is not to say that αsyn cannot be implicated in such alternate cell death mechanisms. For example, as previously discussed, αsyn can be taken up into microglia and induce the production and excretion of inflammatory mediators.

A final challenge to the prion-like propagation of αsyn pathology accounting for PD is the observation that Lewy pathology is not necessary for nigral degeneration and the clinical presence of parkinsonism. While pathological studies on genetic forms of PD are limited, it is clear that at least some of these patients do not show classic Lewy pathology. Indeed, in the case of LRRK2 mutations even within a single family, a spectrum of pathology has been observed among family members with manifesting PD, ranging from those completely lacking any synuclein pathology, to those with significant αsyn aggregation [80-84]. Furthermore a recent study comparing neuronal dysfunction in relation to Braak stage in patients with PD, individuals with ILBD and controls has demonstrated that both cellular dysfunction (loss of tyrosine hydroxylase) and neurodegeneration can precede Lewy body pathology in ILBD [73]. These findings are in direct contrast to those of Luk and colleagues [47] who suggest quite the reverse, that synuclein pathology precedes cell loss. Clearly, further studies will need to be performed to address these concerns.

## Conclusions

Recent years have seen a tremendous surge of interest in the possibility that neurodegenerative diseases, including PD, could develop and progress via non-cell autonomous means, spreading by transcellular mechanisms with seeding and subsequent permissive templating, in a prion-like fashion. If these mechanisms are indeed integral to the pathogenesis of PD and other diseases, this could have important therapeutic implications, as recently discussed in more detail elsewhere [85,86]. These possibilities include treatments designed to block the release or uptake of the pathogenic protein, increase its extracellular clearance by microglia, inhibit protein assembly by, for example, nanomaterials [87] and increase degradation of aggregates, for example, by lysosomes. Currently available drugs may have additional inhibitory effects on the endocytosis mechanism that mediates the internalization of αsyn oligomers [88]. Another approach that could be available for clinical study in the near future might be the use of monoclonal antibodies or immunization therapy, which might be expected to have greater potential for benefit if there is indeed an extracellular phase of αsyn transmission. In light of the fact that there is currently no available therapy to slow or halt the pathological progression of PD, the potential therapeutic implications of a therapy targeted at the mechanism responsible for the transmission of pathology from one region to another are formidable. Thus, it is vital that further *in vitro* and *in vivo* studies are performed to validate the potential involvement of αsyn as a prion-like factor and tease out the mechanisms by which αsyn may be responsible for disease progression in the human condition. However, in view of the unresolved challenges highlighted in this review, caution should be taken in uncritically accepting a role for a prion-like process, particularly in all cases of PD. Indeed, the mechanisms contributing to the progression of the disease may be as variable as the disease itself.

## Abbreviations

PD: Parkinson’s disease; αsyn: Alpha synuclein; DMV: Dorsal motor nucleus of the vagus nerve; CNS: Central nervous system; PNS: Peripheral nervous system; ENS: Enteric nervous system; AGEs: Advanced glycation end products; RAGEs: Receptors for advanced glycation end products; GFP: Green fluorescent protein; MCNSCs: Mouse cortical neuronal stem cells; ILBD: Incidental Lewy body disease; PDD: Parkinson’s disease dementia; DLB: Dementia with Lewy bodies.

## Competing interests

NPV, PLB & L-NH declare no conflicts of interest. AEL has served as an advisor for Abbott, Allon Therapeutics, Astra Zenica, Avanir Pharmaceuticals, Biovail, Boerhinger-Ingelheim, BMS Cephalon, Ceregene, Eisai, GSK, Lundbeck A/S, Medtronic, Merck Serono, MSD, Novartis, Santhera, Solvay, and Teva; received grants from Canadian Institutes of Health Research, Dystonia Medical Research Foundation, Michael J. Fox Foundation, National Parkinson Foundation, Parkinson Society of Canada, and Ontario Problem Gambling Research Centre; received publishing royalties from Saunders, Wiley-Blackwell, Johns Hopkins Press, and Cambridge University Press; and has served as an expert witness in cases related to the welding industry.

## Authors’ contributions

NPV & PLB carried out literature searches, assisted in generation of Figures and writing of the manuscript. AEL & L-NH assisted in writing of the manuscript. All authors read and approved the final manuscript.
